# Food Waste Biorefinery: Pathway towards Circular Bioeconomy

**DOI:** 10.3390/foods10061174

**Published:** 2021-05-24

**Authors:** Bahiru Tsegaye, Swarna Jaiswal, Amit K. Jaiswal

**Affiliations:** 1 Department of Food Process Engineering, College of Engineering and Technology, Wolkite University, Wolkite 07, Ethiopia; bayalew@ch.iitr.ac.in; 2 School of Food Science and Environmental Health, College of Sciences and Health, Technological University Dublin—City Campus, Central Quad, Grangegorman, D07 ADY7 Dublin, Ireland; swarna.jaiswal@TUDublin.ie; 3 Environmental Sustainability and Health Institute (ESHI), Technological University Dublin—City Campus, Grangegorman, D07 H6K8 Dublin, Ireland

**Keywords:** biorefinery, platform chemicals, biofuels, biopolymer, bio-based materials, sustainable production, sustainable development goal

## Abstract

Food waste biorefineries for the production of biofuels, platform chemicals and other bio-based materials can significantly reduce a huge environmental burden and provide sustainable resources for the production of chemicals and materials. This will significantly contribute to the transition of the linear based economy to a more circular economy. A variety of chemicals, biofuels and materials can be produced from food waste by the integrated biorefinery approach. This enhances the bioeconomy and helps toward the design of more green, ecofriendly, and sustainable methods of material productions that contribute to sustainable development goals. The waste biorefinery is a tool to achieve a value-added product that can provide a better utilization of materials and resources while minimizing and/or eliminating environmental impacts. Recently, food waste biorefineries have gained momentum for the production of biofuels, chemicals, and bio-based materials due to the shifting of regulations and policies towards sustainable development. This review attempts to explore the state of the art of food waste biorefinery and the products associated with it.

## 1. Introduction

The environmental problem is one of the most difficult issues challenging the world today. The fast-growing world population accelerates the need for food and other basic materials, which is accompanied by the bulk generation of waste biomass. This directly contributes to the increased cost of waste disposal and causes significant environmental problems. The growing population is directly proportional to the increased demand of food and subsequently the larger quantity of food production that is accompanied by bulk generation of food wastes. According to the United Nations Food and Agricultural Organization (FAO), 1/3 of the total food produced was lost in the supply chain and harvesting which contributed to the estimated value of USD 1 trillion annual loss [1]. The drink industries are leading by generating around 26% of the total food waste, followed by the dairy industry which contributes 21%, fruit and vegetable industry 14.8%, and cereal industry 12.9% [2]. Other than the economic impact, food waste is a potent greenhouse gas emitter (mainly methane) contributing to environmental pollution. More recently, food waste is directly connected to water loss, air pollution, water pollution, biodiversity loss, soil degradation, and climate change. The loss of food as waste which was intended to be for human consumption is likely to be linked to nutritional loss in diet.

Food waste includes spoiled foods, crops left in the field, fruit and vegetable waste, leftovers on the plate from hotels, homes, and restaurants, and any other food lost at any stages of the supply chains. It is impossible to completely avoid food waste, however it is possible to reduce the amount of wasted food. Therefore, crafting ways or methods of valorizing food waste are crucial for developing sustainable bioeconomy and for achieving United Nations (UN) sustainable development goal of 2030 [3]. Due to their homogeneity, food waste has high potential for the production of biofuels, platform chemicals and bio-based materials by applying the concept of biorefinery [4,5]. The valorization of food waste under the biorefinery framework has recently gained momentum for the implementation and achievement of the sustainable development goals policies set by the European Unions (EU), such as the bioeconomy strategy and the circular economy goals of the EU [6,7,8]. According to the bioeconomy council, “The bioeconomy is the knowledge-based production and use of biological resources to provide products, processes and services in all economic sectors within the frame of a sustainable economic system”. The European bioeconomy strategy focused on the needs of the sustainability and circularity of processes and products [6]. The European commission defined the circular economy as the elimination/minimization of waste generations during the processing and production of products, materials and resources by maintaining the value of the product as long as possible [7]. The concept of the circular bioeconomy is described as the production of energy, food, platform chemicals, and other bio-based materials and compounds from biomass in a sustainable and integrated/cascaded manner (biorefinery) while generating zero waste [6,7].

Europe was the first continent to step up crafting policies and strategies for the sustainable production of materials and chemicals by minimizing and eliminating food waste. The policies and regulations forced many industries to reconsider their ways of productions and started shifting towards greener technologies. Therefore, converting food waste into biofuels, bio-based fertilizers, bio-based enzymes, chemicals, proteins and other bio-based molecules and materials will accelerate the sustainable development goals. Moreover, it has the advantages of: (i) achieving the goals of zero waste generations; (ii) reducing/eliminating waste management problems; (iii) reducing/eliminating waste management related costs; (iv) helping the sustainable production of materials and chemicals; (v) fostering the circular bioeconomy. Therefore, employing green technologies for recovering more valuable products from food waste helps to reduce environmental problems.

The shifting of policies and regulations is forcing the minimization of waste generation and it encourages the bio-based economy. The integration of processes that produce products and materials in a more circular and sustainable way is the only possible scenario for food waste valorization that achieves the sustainable development goals. Moreover, comprehensive studies on the recovery of multiple products are mandatory to tackle the current challenges of food waste biorefinery, and numerous articles have been published in this area. In this article, we have systematically reviewed the state of the art of food waste biorefineries. The article critically evaluates the recent research focused on food waste biorefineries employed to produce biofuels, platform chemicals, biopolymers, bio-based fertilizers, bio-based enzymes, proteins, and other bio-based molecules and materials. Furthermore, the transition from the linear economy to a more circular economy by achieving sustainable development goals has been assessed. The technological hurdle for achieving zero waste policy are discussed and possible scenarios were explored.

## 2. Food Waste Generations

Food waste includes both the edible and non-edible parts of food that are generated throughout the whole chains of food supply. The United Nation’s SDGs have targeted a 50% reduction in food waste by 2030 [3]. According to the UN Environment Program Food Waste Index report of 2021, about 931 million tons of food waste were generated across the globe in the year 2019 [9]. Approximately, 40% of the total food produced in the world are wasted along the supply chains. The figure is quite different from region to region and in supply chain stages. In developing countries (low-income countries), a significant amount of food was wasted in the pre-harvest and post-harvest stage while in the developed nations it was wasted in the consumption stage [10,11]. The total amounts of food waste generated by countries across the globe are shown in Table 1 [9].

According to the US Environmental Protection Agency (EPA) estimates, about 63.1 million tons of food waste was generated in United states in the year 2018 [12]. The EU generates around 88 million tons of food waste (estimated monitory value of EUR 143 billion) annually where house hold accounts about 70% of the total waste [13]. The food waste generation in Europe and in the global scale ranges from 158 kg/person/year to 298 kg/person/year and 194 kg/person/year to 389 kg/person/year, respectively [14]. There are significant gaps of food waste data in the developing countries, and even many countries do not have national statistics for food waste. Even China, the second economic power-house and the world’s most populous nation has no official food waste statistics other than some reports of food waste such as in some schools [15] and restaurants in selected cities [16]. France alone generates around 5.8–9 million tons of food waste annually, which is 20–30 kg/year/person [17,18]. This shows that a significant amount of food is wasted annually unnoticed, which could have been alleviating global poverty. Moreover, it creates huge financial losses, material loss, and more importantly causes environmental pollution. 

## 3. Impact of Food Waste on the Environment

Food waste causes a significant amount of socioeconomic and environmental costs, and the recovery of this resource could have a huge positive impact on the environment and society. In the developed nations, food waste is associate with consumer’s behaviors; while it is associated with the lack of technological incapability in developing nations. According to a US Department of Agriculture report, 30% of food was wasted at the consumer and retail levels, which is about 66.5 million tons, causing a financial loss of USD 161 billion annually [19]. About 95% of this food waste ended up in landfill, which causes a significant amount of anthropogenic methane emissions—about 113 million tons of carbon dioxide equivalence annually [19,20]. This action, which causes environmental pollution and significant health, material and financial losses, is avoidable. Initiatives like food waste prevention intervention campaigns are creating awareness in the consumer spectrum and the results are promising [21]. Around 27.85% reduction in food waste were reported in Arizona by creating awareness through educational interventions [21]. Behavioral effectiveness was also observed in household food waste prevention via psychological based intervention [22]. Worldwide campaigns are needed to promote food waste preventions. However, preventing food waste through campaigns is not very effective and finding ways of utilizing the food waste can significantly reduce the financial loss, material loss, health effects and environmental consequences. France is recovering products such as biogas and bio-based plastic from food wastes after the implementation a food waste valorization policy [17]. Implementing the core principles of the circular bioeconomy is the best way to alleviate the problems associated with food waste.

## 4. Food Waste Biorefinery

Food waste biorefinery is a process by which a broader ranges of food wastes are converted into biofuels, platform chemicals and bio-based materials. For food waste valorization, it is essential to know the compositions, the interaction of its components, and the desired final products for choosing an efficient biorefinery process [23]. In general, food waste biorefinery processes are categorized into three major groups: (i) biological pathway: a process by which food wastes are converted into value added product via enzymes or microorganisms; (ii) thermochemical process: a process by which food wastes are treated at elevated temperature using chemicals as a solvent. This includes liquefaction, pyrolysis, and gasification; (iii) chemical process: a process by which chemicals are used as a solvent and as a catalyst in food waste valorizations. The combination of two or more of the above processes in an integrated manner has been attracting the attention of many researchers due to higher conversion efficiencies.

### 4.1. Bioconversion Processes

#### 4.1.1. Anaerobic Digestion

Anaerobic digestion is a biological process by which organic matters are metabolized and transformed by complex reactions into biogas in the absence of oxygen [24,25]. Anaerobic digestion is commonly found in nature such as in animal digestive system, in swaps and wetlands. Anaerobic digestion is most-commonly practiced throughout the world in many ways, such as the digestion of primary and secondary sewage sludge, upflow of anaerobic sludge blanket reactors, and activated sludge plants [26,27,28]. The process consists of four steps: hydrolysis, acidogenesis, acetogenesis, and methanogenesis, which may occur sequentially or simultaneously in a single stage. Products such as methane (CH_4_), volatile fatty acids (VFAs), such as propionic acid, butyric acid, acetic acid, iso-butyric acid, valeric acid, iso-valeric acid, and hydrogen (H_2_) are produced from food waste via anaerobic digestion or anaerobic fermentation.

The anaerobic digestion can be either performed in single stage or two stage operations. In the single stage configuration, all reactions are carried out in a single reactor that helps toward low operational costs and low reactor complexity. However, the formation of intermediate products accelerates inhibition of the subsequent processes. Hence, the lower conversion efficiency and lower product yield are obtained in such reactor configurations. Generally, in single stage reactor configurations, process instability, reactor acidifications, and the combined production of hydrogen and methane are common problems [29,30]. The two-stage process in which the acidogenic and methanogenic processes are physically separated appears to be effective, overcoming problems associated with single stage digestion [31]. The anaerobic digestion of mixtures of food waste, poultry litter, and sewage sludge enhanced the biogas yield to 640 L/kg VS when mixed in the ratio of 2:1:1, sewage sludge: food waste: poultry litter [32]. The anaerobic digestion of food waste for methane production at mesophilic temperature (34 °C) generated 276.5 mL CH_4_/g VS while 307.5 mL CH_4_/g VS was obtained at the thermophilic temperature of 55 °C [33]. A study carried out by Patinvoh et al. [34] observed that the yield of VFAs was enhanced by controlling the pH of acidogenesis process (at pH 6) during the anaerobic digestion of food waste [34]. The highest yield of VFA (0.8 g VFA/g VS) was achieved at an inoculum to substrate ratio of 1:3 [34]. The integration of dark fermentation (acidogenesis) and methanogenesis of food waste enhanced the biohythane (H_2_ + CH_4_) production by 1.22 times [35]. The yield of methane from the anaerobic digestion of one ton of food waste can be as high as 90.6 m^3^ [36]. The reaction configurations of anaerobic digestion are highly influenced and controlled by process parameters (pH, acidity, temperature, substrate composition, C/N ratio, reaction time and inoculum) and the desired final product. Therefore, optimizing the process parameters enhances the yield of the desired final product.

#### 4.1.2. Dark Fermentation

Dark fermentation is a microbial conversion process in which hydrogen is produced by anaerobic bacteria from organic matters via glycolysis pathway. It is performed in the absence of light by a diverse group of bacteria. The cost effectiveness and the possibilities of utilizing wide ranges of substrates in the dark fermentation for biohydrogen production have been studied by numerous authors [37,38]. However, problems associated with low hydrogen yield and high cost of production is a challenge for scale up and commercialization of dark fermentation technology [38]. Theoretically, 12 moles of biohydrogen are expected from one mole of glucose, however maximum yield of four moles of biohydrogen were obtained when acetic acid was the end product, while two moles were produced when butyric acid was the end product. With VFAs formations, 2–3 moles of biohydrogen are obtained from one mole of glucose [39]. Dark fermentation of food waste collected from cafeterias yielded 1.77 moles of H_2_/mole of hexose [40]. However, sequential dark fermentation and photofermentation increased the biohydrogen production by 2.5-folds using 5.4-moles of H_2_/mole of hexose [40]. Dark fermentation using food waste at the mesophilic temperature of 34 °C led to a biohydrogen yield of 53.5 mL H_2_/g VS, while 37.6 mL H_2_/g VS were obtained at the thermophilic temperature of 55 °C [33]. Nguyen et al. [41] studied the single stage dark fermentation of food waste mixed with condensed molasses to produce biohythane (H_2_ + CH_4_). Biogas comprising 10–60% H_2_, and 5–20% CH_4_ was obtained depending on the ratio of food to microorganism [41]. The co-existence of a wide range of microorganisms can significantly reduce the yields of biohydrogen by either utilizing the produced biohydrogen or metabolizing the substrate into other products [42]. The operating conditions highly influence the specific microbial communities and the final product. Acetate and butyrate pathways are linked to higher biohydrogen yields while alcohol and lactate production pathways are linked with lower biohydrogen yields [43,44]. Optimizing the fermentation conditions significantly enhances the biohydrogen yield but is far from reaching the near theoretical yield. Adjustment of the reactor configurations for utilization of the intermediate products during co-culturing or sequential photofermentation can greatly enhance the biohydrogen yield. Metabolic engineering has great potential to alter the current barriers of dark fermentation, and the application of metabolic engineering principles to the selected strains of microorganisms has a promising future, which could revolutionize the whole biorefinery process.

#### 4.1.3. Electro-Fermentation

Electro-fermentation is a new type of hybrid technology that combines the old fermentation principles and electromicrobiology for the improvement of product yields. It uses polarized electrodes to redirect the transfer of small number of electrons into and/or from the medium. The main source of electrons during the electro-fermentation process is the organic material in the medium, because the number of electrons exchanged at the polarized electrode is low compared to the microbial electrosynthesis [45,46,47]. The interactions of the microorganisms with the electrode during electro-fermentation are either through DIET (direct interspecies electron transfer mechanisms) or MIET (indirect interspecies electron transfer mechanisms) [48]. The electron transfers are achieved by mediators/shuttles produced by cells such as flavins, formate, phenazines, and H_2_ in case of MIET while electrically conductive pilus or proteins such as cytochromes are used in case of DIET [48,49,50]. *Shewanella oneidensis* and *Geobacter sufurreducens* are the two most commonly studied electroactive bacteria and are considered as a model for DIET. This impressive capability observed in some bacteria can be exploited for biohydrogen production. Recently, electro-fermentation has been employed on food waste valorization and promising results were obtained [51,52,53]. About 26.3% improvement in the methane production was achieved by limiting the amount of volatile fatty acids to 129 mg/L from the electro-fermentation of food waste [52]. Hydrogen recovery was also improved by the sequential process of electro-fermentation of food waste from the effluents of dark fermentation [53]. Therefore, further studies are required to fully exploit the microbial potential for biohydrogen production as well as for other biomaterials from different food wastes.

#### 4.1.4. Photofermentation

Photofermentation is a fermentation process in which light is used as an additional source of energy. The purple non-sulfur bacteria (PNSB) are the most common photosynthetic bacteria. Electrons are driven out from the organic food waste by nitrogenase enzyme of the photosynthetic bacteria to produce carbon dioxide and hydrogen [54]. Food wastes such as glycerol that contain simpler organic compounds and short chains fatty acids are ideal substrates for photofermentation [54,55]. Photofermentation as a green technology has a great potential and capability for production of biohydrogen from food waste as evident from wastewater treatments emerging from industries such as dairies [56], distilleries [57], brewery [58], and sugar refinery [59]. The production cost of 1 kg of hydrogen by photofermentation was estimated to be about EUR 2.83, while electrolysis-based technology costs from EUR 4–24 [60]. The presence of inhibitory compounds in the waste, lower light penetrations due to the turbidity of the waste, and the rate of cell wash out exceeding the specific growth rates are some of the major challenges hindering the production of biohydrogen by photofermentation [61,62]. The immobilization of microbial cells is an effective approach to overcome the over washing, while other drawbacks need to be resolved [63]. To use the full power of photofermentation, the drawbacks have to be resolved. Therefore, intensive research is required to develop feasible and sustainable photofermentation technology to utilize food waste for high-value products production.

### 4.2. Integrated Approach

Integrated approaches are considered in order to improve the economics of food waste treatments, enhancing product yields, and reducing the current high production costs. Two stage dark fermentation integrated with microalgal cultivation (MC) was applied to improve overall energy and resource recovery [64]. Enriching starchy waste-water with poultry manure to increase the nitrogen supplement in dark fermentation enhanced the biohydrogen yield from 4.11 mol/kg COD (chemical oxygen demand) to 5.03 mol/kg COD, while the remaining spent was utilized for biodiesel production by *Chlamydomonas reinhardtii* [64]. On the other hand, thermal pretreatments (at 121 °C for 15 min) of starch wastewater enriched with groundnut de-oiled cake showed an improved biohydrogen production of 3.24 L/L and biohydrogen yield of 12.05 mol H_2_ kg^−1^ COD [65]. The addition of nano-metal oxides in rice mill wastewater during dark fermentation by *Clostridium beijerinckii* DSM 791 showed improved biohydrogen production, while the addition of NiO and CoO nanoparticles enhanced biohydrogen yields by 109% and 90% respectively [66]. The integration of dark fermentation and photofermentation significantly improves the biohydrogen yield. In this hybrid system, biohydrogen and organic acids are produced during dark fermentation and enhanced biohydrogen were produced by dark fermentation using purple nonsulfur bacteria [67,68]. The mode of operation of this hybrid system is either in a single stage (combined system) or sequential (two stage), and was found to be very efficient for biohydrogen production. The two-stage system (sequential) is more promising, as the metabolic products of dark fermentation sometimes require treatment and different optimal conditions [69]. The overall reaction of integrated dark fermentation and photofermentation in a sequential manner is:

$$C_{6}H_{12}O_{6}+2H_{2}0\to 2{\mathrm{CH}}_{3}\mathrm{COOH}+2{\mathrm{CO}}_{2}+4H_{2}\;\left(\mathrm{dark}\;\mathrm{fermentation}\right)$$

$${\mathrm{CH}}_{3}\mathrm{COOH}+2H_{2}O\to 4H_{2}+2{\mathrm{CO}}_{2}\;(\mathrm{photofermentation})$$

**Figure 1:**
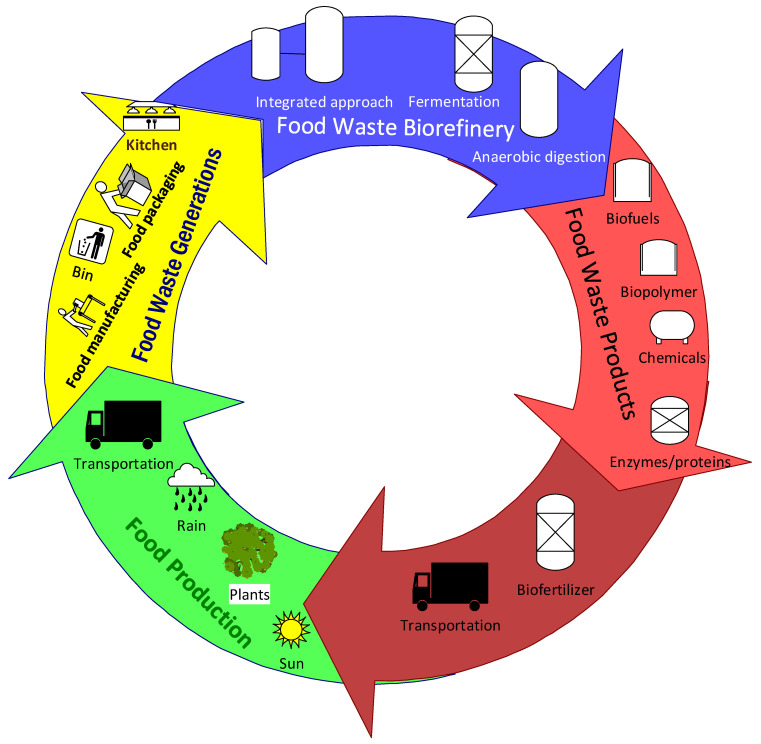
The prospect of circular bioeconomy in food industries.

These show the potential of the integrated food waste biorefinery process for opening up the way for the circular economy. More investigations and research studies on how to improve the efficiencies of conversion and product yield in the pilot scale and commercial scale are key for the transition to bioeconomy. The overall complexity of the food industries and the relationships with the circular bioeconomy and sustainability are described in Figure 1.

## 5. Food Waste Biorefinery Products

Food waste biorefinery is considered as a promising technology to valorize waste and minimize environmental challenges through efficient utilization of resources. The products obtained from waste via biorefinery will minimize fossil-fuel dependency and switches towards circular economy. Numerous products such as protein, animal feed, enzymes, organic acids, flavors and colorants, bio-fertilizers, bioplastics and biofuels can be produced simultaneously and sequentially from food waste by applying the concept of biorefinery. Some of the potential products produced from food waste biorefineries are discussed below.

### 5.1. Biofuels

Biohydrogen (H_2_), methane (CH_4_), and bioethanol (CH_4_CH_2_OH) are the main final products of organic polymer degradation (food waste) of microbial metabolites. Higher yields of biohydrogen were observed after volatile fatty acids yields were improved by electro-fermentation [70]. They observed biohydrogen yields of up to 26% with volatile fatty acids recovery of 4595 mg/L from food waste by electro-fermentation [70]. Increased biohydrogen and volatile fatty acids yields were observed by calculating salinity level up to 40 g/L of NaCl [71]. The addition of NaCl favored the production of butyric acid and inhibited the methanogenesis process while favoring the acidogenesis process that contributed for higher biohydrogen production [71]. Enhancement of CH_4_ and biohydrogen production was also absorbed from food waste collected from restaurants. About 0.61 L/g VS of biohydrogen and 0.42 L/g VS of CH_4_ were produced in a sequential hydrolysis of carbohydrate rich food waste collected from restaurants in acidified leach bed reactors and methanogenic reactors [72]. Immobilization of bacteria further enhanced the production of biohydrogen. The continuous production of biohydrogen from popular biomass hydrolysate showed improved biohydrogen yield of about 2.83 mole H_2_/mole of hexose which were observed over a 40-day period, that was four-fold higher than the best biohydrogen producing strains, *B. thuringiensis* [73]. The complete valorization of date byproducts (inedible and discarded part of date fruit) resulted in 292 mL H_2_/g VS and 235 mL CH_4_/g VS accompanied with date syrup production via hot water extraction of the byproduct, which resulted in syrup content of 35.5% sucrose, 11.8% glucose and 13.17% fructose [74]. Promising results were observed from scaling up of biohydrogen production from organic spent matters in batch process. Biohydrogen yield was increased from 46 mmol H_2_/L to 73 mmol H_2_/L (1.5-fold increase) by scaling up from lab scale to pilot scale (13.5 L) at regulated pH and reduced partial pressure conditions from molasses spent by the *Clostridum butyricum* TM-9A strain [75]. The complete valorization of the date biomass is one illustration of a biorefinery approach for waste biomass conversion to bioenergy, platform chemicals and other bio-based materials. Various types of products are produced from different food waste types. The various types of biofuel obtained from different types of food wastes are summarized in Table 2.

### 5.2. Platform Chemicals

Short chain fatty acids/volatile fatty acids are essential industrial chemicals used for the production of acidulant, flavoring agents, polymers, preservatives and many other applications in food industry, pharmaceutical industries, and cosmetic industries [85,86]. Co-fermentation of food waste and waste activated sludge (WAS) was tested experimentally for VFAs, carboxylic acid and lactic acid productions. A wide ranges of platform chemicals are extracted and produced from various types of food wastes (summarized in Table 3). The result of co-fermentation (WAS/food waste_50/50) profile shows that 47% butyric acid, 19% valeric acid and 18% acetic acid at day 6 and pH 5.3, while 40% acetic acid, 26% butyric acid and 15% propionic acid at pH 4.3 during the same fermentation period and conditions [87]. They observed pH affects the concentration of acetic acid and lactic acid and lower pH favors their accumulations [87]. VFAs filtration inhibits methanogenesis of food waste in the bioreactor [88]. A continuous recovery of VFAs (highest yield of 0.54 g VFA/g VS) from food waste by anaerobic immersed membrane bioreactor was developed [89]. The VFAs yield was enhanced by regulating acidogenesis of anaerobic digestion by electro-fermentation of food waste [70]. About 4595 mg/L of VFAs was recovered from food waste after external stimulation of fermentation broth by electron [70].

Carboxylates are produced by a sequential process of hydrolysis and acidogenesis of food waste. Hydrolysis disintegrates the larger polymers such as carbohydrates, proteins, and lipids into smaller chain monomers such sugars, long chain fatty acids and amino acids. The next stage, acidogenesis completes the formation of carboxylates and biogas from hydrolyzed polymers. A high amount of lactic acid (52 g/L) was produced by dark fermentation after enzymatic pretreatment and controlling the total solid content of food waste at 34% [90]. Recently, the attempt to recover medium chain carboxylic acids by granular chain elongation process from waste biomass was observed to be promising [91]. They achieved maximum yield of 72.86% of medium chain carboxylic acids by adding ethanol and CO_2_ (at a loading rate of 2 L/d) at 2.5-day hydraulic retention time of sludge fermentation broth [91]. The CO_2_ supply facilitated oxidation of ethanol to acetyl-CoA by lowering the partial pressure of hydrogen [91]. Carboxylic acid yield of 0.62 mg/mg COD_A_ was achieved from glycerol rich food waste [92]. Production of caproic acid was enhanced by ultrasonic pretreatment (207.8 mg COD/g VS) and hydrothermal pretreatments (210.1 mg COD/g VS) of food waste compared with alkali thermal pretreatments during acidogenic fermentation by *Caproiciproducens* [93]. Besides VFAs and carboxylic acids, a range of chemicals are simultaneously recovered from food waste biorefinery. Phosphorus, vivianite and VFAs were simultaneously recovered from WAS and food waste co-fermentation [94]. Enhanced recovery of phosphorus (83.09%), vivianite (93.9% purity), and VFAs (7671 mg COD/L) from 30% food waste and 70% WAS with variable pH caused by microbial activity were obtained [94]. The conversion technology of waste biomass into platform chemicals are rapidly evolving. This is mainly due to the shifting of polices and regulations from linear economy to circular economy in many countries and regions across the globe. Therefore, further research and investigations in the technologies of waste conversions to platform chemicals, biofuels, and materials are vital to sustain life in our planet.

### 5.3. Biopolymers 

Food waste are rich in carbohydrates, and proteins and are potential sources of biopolymers. The biopolymers have especial advantages in the domain of biodegradable packaging materials. Wastes from fish processing industries are rich in biopolymers such as chitin, collagen, chitosan, and gelatin which have prominent application in novel food packaging technologies. Biopolymers such as polysaccharides, polyhydroxyalkanoates (PHAs), aliphatic polyesters and polylactides have potential application in the transformation from fossil fuel-based plastic to bioplastic production. Sugar rich food waste such as lignocellulosic biomass, whey, legume wastes, sugar wastes, whey, and oil are also important resources for PHAs production via bacterial hydrolysis and fermentation. About 66% PHBV were produced by pure culture of *Haloferax mediterranei* from whey in a fed batch fermenter [104], while 61.5% were achieved from cassava starch by *Cupriavidus* sp. KKU38 strain [105]. Gelatin or myofibrillar proteins extracted from fish wastes are low-cost substrates for bioplastic productions [106,107]. The production of biopolymers from food waste is an opportunity for minimizing the environmental impacts and is a way of moving towards circular economy. Various biopolymers from food wastes such as PHAs, polybutylene adipate terephthalate (PBAT), polyhydroxybutyrate (PHB), polylactic acid and polyesters have been identified and investigated and promising results are obtained [108,109,110]. This shows that the potential application of biorefinery concept for valorizations of food wastes into variety of products. Therefore, this can not only achieve the goals of sustainable development and productions but also reduces production costs of materials and chemicals significantly.

### 5.4. Bio-Based Proteins and Enzymes

Microorganisms grow on various substrates and are potential sources of low-cost alternative media for cultivation of microorganisms in order to produce products of industrial interest. The metabolic products and the microorganism itself are the source of many proteins and enzymes. Single cell proteins can be obtained by harvesting and drying the microbial biomass [111]. It is also termed as microbial protein and is produced most commonly by submerged fermentation and solid-state fermentation [112]. Solid-state fermentation of whey, orange and potato residues, molasses, brewer’s solid waste by *K. marxianus* IMB3 (thermotolerant), Kefir culture and *S. cerevisiae* AXAZ-1 (psychrotolerant and alcohol resistant) were used to produce aroma compound pinene, protein, and lipid [113]. The optimal growth condition for *K. marxianus* IMB3 was 30 °C and pH 7 and kefir culture and *S. cerevisiae* AXAZ-1 was 30 °C and pH 5.5 [113]. Kefir culture produced about 4 kg of the aroma compound pinene per ton of the food waste while *S. cerevisiae* AXAZ-1 produced 38.5% protein [113]. Yunus et al., produced a single cell protein by growing *Candida utilis* and *Rhizopus oligosporus* on wheat bran [114]. A protein yield of 41.02% was obtained at optimal fermentation conditions of 30 °C and 48 h [114]. The metabolite analysis of cultivation of microalgae *Aphanothece microscopca nageli* on rice effluent shows a high yield of single cell protein and high ratio of polyunsaturated fatty acid (mainly gamma linolenic acid) [111]. Protein with essential amino acid content, such as threonine, lysine, valine, and leucine was obtained after solid state fermentation of yam peel for 96 h by *Saccharomyces cerevisiae* BY4743 [115]. Single cell protein is a good source of essential amino acids and has a potential of bulk production within short time, hence it may replace expensive sources of protein [116].

Protease and esterase enzymes are extracted from fish wastes have potential applications in industrial and medical industries. Protein yield of 55.15% was obtained by isoelectric-ammonium sulfate precipitation method from sugar beet byproduct [117]. Valorization of shrimp waste by *Haloferax lucentensis* GUBF-2 MG076078 produced high protease enzyme (101.98 U/mL) while highest lipase enzyme (5.83 U/mL) was produced from coconut oil cake at optimal conditions, pH 6, NaCl 30% and temperature 42 °C [118]. The yield of pectinase enzyme was enhanced by reduced fatty acid biosynthesis and further increased by inhibition of pyruvate dehydrogenase and fatty acid biosynthesis by furfural and triclosan [119]. High amylase enzyme activity (29.23 mg/mL) was reported on mango waste using *Bacillus* sp. F-11 bacteria [120]. Various types of proteins and enzymes that are extracted from food waste biomass are summarized in Table 4. These results show the potential application of food waste for extracting and isolating vital enzymes and proteins from food waste.

### 5.5. Bio-Based Fertilizers

Bio-based fertilizers improve the physico-chemical properties of soil and can help to reduce the amount of waste disposed, benefiting the environment. Composting is the most common practiced method of food waste recycling for the purpose of bio fertilizer production due to easy of storing, handling and transportation [121]. However, the unstable conditions created due to dynamics of environmental factors, pH, temperature, type and content of food waste makes difficulty of maintaining stable degradation process. The quality of biofertilizer was improved (nitrogen content was increased from 2.01% to 2.10%, ash content from 24.94% to 29.21%) after microbial degradation of food waste by *Brevibacillus borstelensis* SH168 thermophilic and lipolytic bacteria [122]. Thermal hydrolysis of food waste produced liquid organic fertilizer by removing the biotoxicity and phytotoxicity of the liquid fertilizer [123]. The micronutrients (Fe, Cu, Zn, Al, Co and Mn) of the biofertilizer were significantly improved with higher nitrogen (1685 mgN/L) and phosphorous (235 mgP/L) content with potassium content unchanged at a 180 °C of thermal treatments [123]. High purity phosphorous (81%) from waste were recovered by electrodialysis and 74% of nitrogen in the form of nitrate was recovered from waste by gas permeable membrane for production of biofertilization [124]. The sequential digestion of the two-stage anaerobic process followed by the aerobic process of fruit and vegetable waste mixed with slaughterhouse waste significantly enhanced biofertilizer formations [125]. The process generated 29.2 L/kg of biogas from the anaerobic digestion of fruit and vegetable waste and biofertilizer of C:N ratio of 10:11 [125]. Biofertilizer can improve soil fertility, maintain the natural ecosystem, and help to reduce the environmental impact caused by food waste, contributing toward the green economy. Therefore, much work is needed to recycle and reuse food waste for achieving the bioeconomy.

### 5.6. Other Bio-Based Compounds and Materials

Bioactive compounds are one of the high commercial value products and are extracted from a variety of plant-based resources. However, the extractions from food waste, especially plant-based food waste, have been attracting greater interest in recent years. It increases the economic significance of food waste. Phenolic compounds have well known applications in food, medical and pharmaceutical industries due to their antiviral, antibacterial, antioxidants, anti-carcinogenic and anti-inflammatory activities which are widely extracted from food waste by conventional or non-conventional techniques [130,131]. Pectin and essential oils were extracted sequentially from fruit wastes (orange peel) using microwave irradiation [132]. Pectin isolated from the biorefining of orange peel waste after essential oil extraction (1.57%) yielded up to 17.4% (w/w) (about 25% w/w of the total pectin in the orange peel) [132]. Spent coffee waste contains approximately 1–1.5% polyphenols and extraction by aqueous ethanol (20%) with microwave irradiation for 40 s at 80 W effectively extracted 399 mg GAE/g equivalent [133]. The application of pulsed electric field on tomato peel at energy inputs of 5-10 KJ/kg and field strength between 1–5 kV/cm enhanced the lycopene yields by 12–18% [134]. The application of a pulsed electric field with ethanol on potato peel further contributed four about 9% increment of antioxidant activity and 10% increment of phenolic yield [135]. Lycopene, β-Carotene, protein and oil were extracted from tomato waste valorization by applying a biorefinery approach. The application of supercritical CO_2_ extraction yields about 410.5 mg lycopene, 31.5 mg β-carotene from a kg of tomato peels and 27 mg lycopene, 5 mg β-carotene from a kg of tomato seeds [136]. Essential oil and lemon pigment were extracted from lemon peel by microwave assisted extraction. The analysis of lemon essential oil by gas chromatography with flame ionization detector reveals that, about 65% limonene, 14% β-pinene and 10% γ-terpinene were the main components whereas ultra-high performance liquid chromatography shows that the lemon pigment contains about 4.7% eriocitrin, 7.3% diosmin, and 2.65% hesperidin [137]. In a different study, high pressure processing (400 MPa/10 min) of lemon peel resulted in higher polyphenol recovery, sinapic acid recovery of 47.33% in oven dried lemon flavedo and 59.59% in essential oil residues and escultein recovery of 16.85% in oven dried lemon flavedo and 18.31% in essential oil residues [138]. The application of green technologies such as microwave assisted extraction, supercritical fluid extraction, ultrasonic assisted extraction, pulsed electric field extraction for the extraction of bioactive compounds and other co-products from fruit and vegetable wastes have been recently reviewed [139,140]. There are no standard procedures for the extraction of bioactive compounds due to the great variety of food wastes, composition and chemistry of the wastes, chemistry of the bioactive compounds and the extraction conditions and/or parameters [131]. Therefore, developing more effective and efficient extraction techniques for particular bioactive compounds from particular food waste is vital for successfully contributing towards the circular bioeconomy.

## 6. Contributions of Food Wastes for Bioeconomy

The urgent need for the transition from the linear economy (fossil fuel-based economy) to the circular economy requires both sustainable resources and sustainable production of materials and chemicals. In this context, food waste is considered as a potential feedstock for sustainable production of chemicals and materials, which is the core idea of circular bioeconomy. Therefore, food waste has a great potential for empowering bioeconomy. The potentials of producing spectrum of products such as biofuels, platform chemicals, enzymes, proteins, biopolymers, biofertilizer and other bio-based compounds and materials from food waste can ensures sustainability of productions as well as resolve the issues of environmental concern.

The overall production cost of polyhydroxyalkanoate (PHA) from slaughtering waste various between EUR 1.41/kg to EUR 1.64/kg depending upon whether offal is considered as waste or not, with biodiesel as a co-product (EUR 0.97/L) [141]. The payback period is from 3.25 years to 4.5 years, which is in a reasonable period [141]. The valorization of tomato waste by supercritical CO_2_ extraction produced about 437.5 mg of lycopene and 36.5 mg β-Carotene [136].

A study carried out by Cristóbal et al. [8] on the techno-economic and profitability analysis of food waste biorefineries at the European level calculated that if the price of lycopene and β-Carotene are assumed to be EUR 40000/kg and EUR 4000/kg, respectively, the biorefinery would be profitable having up to 56 plants installed across Europe. However, the payback time period should be carefully considered in this assessment (the payback period for other biorefineries in the real world implementation ranged between 3 and 15 years) [8]. Potato waste biorefineries for the production of bioactive compounds were profitable by limiting the number of plants to 28 within Europe and with the bioactive compound price fixed at EUR 300/kg based on biorefinery data obtained from Maldonado et al. [142]. The study of the techno-economic analysis is based on the market stability; however, overproduction is a big concern. Biddy et al. [141], demonstrated the potential of increasing succinic acid production by four-fold reduced the price significantly [143]. The demand for some specialty chemicals would be satisfied by just 5–10 biorefineries and a few biorefineries could satisfy the needs of the high-value pharmaceutical markets [144]. Expanding the market size by considering derivative chemicals is vital. Large markets, such as the polymer industry, which are able to support many facilities are crucial to solve the tradeoff between market volume and high value products.

However, implementing large scale biorefineries is associated with various risk factors such as feed stock price risk, feed stock supply risk, policy risk, market risk, and technological risk [145]. The commercial scale operation of biorefineries directly affects the price of feedstock due to the increased demand of raw materials. The size of the biorefinery and the cost of feedstock are the key factors that determines the cost–effectiveness of biorefinery [146]. Sweet sorghum bagasse biorefinery for the production of bioethanol via co-fermentation of hexose and pentose sugar was found to be expensive relative to the equivalent gasoline price [146]. The economic analysis of wood based biorefineries was found to be not profitable for the production of ethylene (0.1 ton), biomethane (130 Nm^3^), hydrolysis lignin (0.45 ton), and organosolv lignin (0.16 ton) with an operating capacity of 400,000 tons of beech wood per day [147]. However, if the selling price of ethylene is increased slightly, the biorefinery could be economical [147]. Currently, the operation of industrial scale biorefineries is not economically viable compared to fossil fuel equivalents [148]. However, the possibility of producing novel materials will lead to price competitiveness and cost-effectiveness of the biorefineries. Moreover, subsidizing bio-products and carbon tax makes biorefinery more competitive and cost effective.

## 7. Conclusions

Food waste valorizations are still in the infant stage. The challenges posed by the growing amount of food waste dumped into the environment creates opportunities for the production of biofuels, platform chemicals and other bio-based compounds and materials via the biorefinery approach. The variation in the type and composition of food waste is also another challenge. During valorization of food waste, the feedstock composition as well as the desired final product should have to be identified for selecting more efficient and effective paths (selection of input–output-appropriate technology). Waste biorefinery is an ideal concept for the valorization of food waste. The efficiency of the product and the cost of production are the main issues needed to be resolved to realize the integration of food waste into the bioeconomy. The development of innovative ways of intermediate product separation are important to achieve these goals. The integration of food waste into the bioeconomy is an inevitable task for the present and future. Therefore, a comprehensive research on both the potential recovery of high-value products and environmental impact assessments such as lifecycle assessments and techno-economic analyses are vital for large scale implementation. Moreover, working towards the implementation of sustainable development goals across the globe and ensuring these goals via government interventions by crafting policies and legislations on how to mitigate and/or utilize food wastes are vital steps for the transition towards a circular economy.

## Figures and Tables

**Table 1:** Food waste estimates by countries across the globe in the year 2019.

Region	Countries	Annual per Capita Food Wastage (kg/Capital/Year)	Estimated Amount of Total Food Waste Generated (Tons/Year)
Global		121	931 million (17% of total produced)
Africa	Egypt	91	9,136,941
	Sudan	97	4,162,396
	Angola	100	3,169,523
	Burkina Faso	103	2,086,893
	Ethiopia	92	10,327,236
	Ghana	84	2,555,332
	Kenya	99	5,217,367
	Mali	103	2,018,765
	Nigeria	189	37,941,470
	Rwanda	164	2,075,405
	South Africa	40	2,329,228
	Uganda	103	4,546,237
	Zambia	78	1,391,729
Asia	Uzbekistan	91	3,001,868
	China	64	91,646,213
	Japan	64	8,159,891
	Indonesia	77	20,938,252
	Malaysia	91	2,921,577
	Vietnam	76	7,346,717
	Bangladesh	65	10,618,233
	India	50	68,760,163
	Pakistan	74	15,947,645
	Iraq	120	4,734,434
	Israel	100	848,395
	Jordan	93	939,897
	Saudi Arabia	105	3,594,080
Australia	Australia	102	2,563,110
	New Zealand	61	291,759
Europe	Hungary	94	908,669
	Poland	56	2,119,455
	Denmark	81	469,449
	Finland	65	361,937
	Ireland	55	267,073
	Norway	79	423,857
	Sweden	81	812,948
	UK	77	5,199,825
	Greece	142	1,483,996
	Italy	67	4,059,806
	Slovenia	34	71,107
	Spain	77	3,613,954
	Austria	39	349,249
	Belgium	50	576,036
	France	85	5,522,358
	Germany	75	6,263,775
	Netherland	50	854,855
	Switzerland	72	616,037
North America	Canada	79	2,938,321
	USA	59	19,359,951
South America	Argentina	72	3,243,563
	Brazil	60	12,578,308
	Colombia	70	3,545,499
	Ecuador	72	1,258,415
	Mexico	94	11,979,364
	Peru	72	2,354,806
	Uruguay	74	255,909

Source: Food Waste Index [9].

**Table 2:** Production of biofuel from food waste biorefinery process.

Feedstock	Bioprocess Type	Reactor Type/Configuration	Products	Yields	Reference
Food waste	Dark fermentation	Lab-scale fermenter	H_2_	1.25 mol/mol of glucose	[76]
Fruit and vegetable waste	Dark fermentation and anaerobic digestion	Integrated CSTR + anaerobic fixed bed reactor	H_2_ and CH_4_	115.2 L H_2_/kg VS 334 L CH_4_/kg COD	[77]
De-oiled Jatropha waste	Acid pretreatment + fermentation	Lab-scale fermenter	H_2_	86 mL/g of reducing sugar	[78]
Orange peel waste	Ensiling + centrifugation	Freezing + thawing	Bioethanol	120 g/kg TS	[79]
Date byproduct (Deglet-Nour)	Dark fermentation	550 mL Plasma bottle	H_2_	292 mL H_2_/g VS	[74]
Date byproduct (Deglet-Nour)	Anaerobic digestion	550 mL Plasma bottle	CH_4_	235 mL CH_4_/g VS	[74]
Carrot discard juices	Batch fermentation	250 mL flask	Bioethanol	11.98 g/L	[80]
Calcium alginate	Batch fermentation	250 mL flask	Bioethanol	29.9 g/L	[80]
Food waste (fruit and vegetable wastes, dairies waste, manure, blood, leftovers, animal feedstuff)	Anaerobic digestion	45 L CSTR 40 °C, 53 HRT	Biogas (60% methane content)	670 NL biogas/kg VS	[81]
	Anaerobic digestion	45 L Fluidized bed reactor 40 °C, 53 HRT	Biogas, (methane content of 60%)	550 NL biogas/kg VS	[81]
Various food waste	Dark fermentation and second stage anaerobic digestion	Fermenter	Biohythane	CH_4_ (70–90%, *v*/*v*) + H_2_ (10–30%, *v*/*v*	[82]
Kitchen waste	Immobilization of oxidase and glucoamylase	Simultaneous scarifications and fermentations, pH 6.2, 55 °C	ethanol	30 g/L	[83]
Waste cooking oil	Immobilization of lipase	Hydrolysis and esterification	Biodiesel	91.8% fatty acid	[84]

**Table 3:** Platform chemicals and bioactive compounds produced from food waste biorefinery.

Feedstock	Bioprocess Type	Reactor Type/Conditions	Products	Yields	Reference
Orange peel waste	Ensiling + centrifugation	Freezing and thawing	Lactic acid	55 g/kg TS	[79]
Orange peel waste	Ensiling + centrifugation	Freezing and thawing	Acetic acid	26 g/kg TS	[79]
Grape stalk	Solvent extraction		Phenols	4.44 g/kg dry solid	[95]
Seed coat waste of red sword bean	Ultrasound treatment	400 W L/S ratio (29.3 mL/g) 500 °C, 18.4 min	Polyphenols	755.98 µmol Trolox/g	[96]
Mung seed waste	Ultrasound treatment	500 W L/S ratio 35:1 700 °C, 46.1 min	Polyphenols	178.28 µmol Trolox/g	[97]
Gac peel	Microwave assisted extraction	120 W, 25 min	Carotenoid and Antioxidant	262 mg/100 g and 716 µmol/L TE/100 g	[98]
Gac peel	Ultrasound assisted extraction	200 W, 80 min	Carotenoid and Antioxidant	268 mg/100 g and 820 µmol/L TE/100 g	[98]
Jackfruit peel	Ultrasound assisted extraction	500 W S/L ratio 1:15, pH 1.6 60 °C, 24 min	Pectin	Yield, 14.5%	[99]
Pastry and cake waste	Hydrolysis and fermentation	Lab-scale fermenter	Succinic acid (96–98% purity)	0.35–0.28 g/g of substrate	[100]
Tomato processing waste	Ultrasound assisted extraction	600 W 60 °C, 8.61 min	Pectin	Yield, 15.21%	[101]
Tomato processing waste	Ultrasound assisted + microwave extraction	(600 W 60 °C, 8.61 min) + (450 W 85.1 °C, 8 min)	Pectin	Yield, 18%	[101]
Tomato processing waste	Ultrasound assisted + Ohmic heating extraction	(450 W, 10 min) + (60 V, 5 min)	Pectin	Yield, 14.6%	[101]
Blueberries waste (Juice waste)	Pulsed electric field	Energy input, 10 kJ/kg	Anthocyanin	75%	[102]
Grape marc	Microwave assisted extraction	48% ethanol, 1.77 g extract, 10 min	Flavanols	1.21 mg GAE/mL	[103]

**Table 4:** Enzymes and proteins from food waste biorefinery.

Feedstock	Bioprocess Type	Reactor Conditions	Products	Activity	Reference
Brewery waste	Lactic acid fermentation	Flask-500 mL, incubator 37 °C, pH 6.5, 100 rpm, *Lactobacillus delbrueckii*	Protease	145 U/g	[126]
Brewery’s spent grain	Solid state fermentation	Glass petri dishes 25 °C, 6 days, *A. nigerCECt2088*	β-glucosidase	94 U/g	[95]
Brewery’s spent grain	Solid state fermentation	Glass petri dishes 25 °C, 6 days, *A. ibericus*	Xylanase	300–313 U/g	[95]
Brewery’s spent grain	Solid state fermentation	Glass petri dishes 25 °C, 6 days, *A. ibericus*	Cellulase	51–62 U/g	[95]
Wheat bran	Submerged fermentation	30 °C, pH 8, 6 days, *A. niger* KIBGE-IB36	Xylanase	3071 U/mg	[127]
Corncob	Submerged fermentation	30 °C, pH 8, 6 days, *A. niger* KIBGE-IB36	Endo-1,4-β xylanase	1523 U/mg	[128]
Wheat bran	Solid state fermentation	*Aspergillus* sp. 28.62 °C, 3 days, 69.92% moisture, 6.42 log inoculum size	Pullulanase	396.2 U/g	[129]
Carrot discard juice	Batch fermentation	Flask 250 mL, *S. cerevisiae* 35 °C, 3 days	Single cell protein		[80]

## Data Availability

Data sharing not applicable.
